# Differences in osteogenic and apoptotic genes between osteoporotic and osteoarthritic patients

**DOI:** 10.1186/1471-2474-14-41

**Published:** 2013-01-25

**Authors:** Mercè Giner, Mª José Montoya, Mª Angeles Vázquez, Cristina Miranda, Ramón Pérez-Cano

**Affiliations:** 1 Bone Metabolism Unit, Internal Medicine, “Virgen Macarena” University Hospital, Avda. Dr. Fedriani s/n, 41009, Sevilla, SPAIN; 2 Medicine Department, University of Seville, Seville, Spain

**Keywords:** Human bone, Osteoporosis, Osteogenesis, Apoptosis, Microstructural

## Abstract

**Background:**

Osteoporosis is a metabolic disorder characterized by a reduction in bone mass and deterioration in the microarchitectural structure of the bone, leading to a higher risk for spontaneous and fragility fractures.

The main aim was to study the differences between human bone from osteoporotic and osteoarthritic patients about gene expression (osteogenesis and apoptosis), bone mineral density, microstructural and biomechanic parameters.

**Methods:**

We analyzed data from 12 subjects: 6 with osteoporotic hip fracture (OP) and 6 with hip osteoarthritis (OA), as the control group. All subjects underwent medical history, analytical determinations, densitometry, histomorphometric and biochemical study. The expression of 86 genes of osteogenesis and 86 genes of apoptosis was studied in pool of bone samples from patients with OP and OA by PCR array.

**Results:**

We observed that most of the genes of apoptosis and osteogenesis show a decrease in gene expression in the osteoporotic group in comparison with the osteoarthritic group. The histomorphometric study shows a lower bone quality in the group of patients with hip fractures compared to the osteoarthritic group.

**Conclusions:**

The bone tissue of osteoporotic fracture patients is more fragile than the bone of OA patients. Our results showed an osteoporotic bone with a lower capacities for differentiation and osteoblastic activity as well as a lower rate of apoptosis than osteoarthritic bone. These results are related with structural and biochemical parameters.

## Background

Normal bone remodelling requires a balance between bone formation and resorption and disruption of either activity or both leads to metabolic bone disorders, being osteoporosis the most prevalent one. In the process of bone formation and resorption different regulatory systems are involved, mainly communication systems between different bone cells: osteoblasts, osteoclasts and osteocytes [1].

Microarrays studies showed that the expression of several genes is related with BMD and with osteoporotic disease [2]. Bone remodelling is regulated by numerous signalling pathways. It is becoming clear that bone formation and homeostasis require at least six major universal signalling transduction pathways: Notch, Wnt, TGF/BMP, Hedgehog, steroid hormone receptor, and receptor tyrosine kinase [3,4]. Most signalling pathways converge in a few transcription factors such as Runx2, Osterix, Sox9 and AP-1 [5,6].

One of the best known processes of bone regulation is OPG/RANKL/RANK that regulates directly the action of osteoblasts and osteoclasts [7], and changes in the production of osteoprotegerin (OPG) and/or RANKL produces misbalances in bone remodelling which can lead to a major formation or resorption depending on the final balance between both proteins.

Apoptosis plays a critical role during embryonic limb development, skeletal maturation, adult bone turnover by modeling and remodeling processes, and during fracture healing and bone regeneration. In humans, increased osteocyte apoptosis has been correlated with sites of rapid bone turnover [8], and osteoblast apoptosis plays an important role in bone development and maintenance. It is estimated that 60–80% of osteoblasts that originally assembled at the resorption pit die by apoptosis. Further, bone loss caused by sex steroid deficiency, glucocorticoid excess, or aging is caused in part by osteoblast apoptosis [9].

As a result of the balance between bone formation and resorption, bone structure may be modified and thus the biomechanical properties which are determinant of bone strength and fracture resistance.

Therefore, in the present study, we determined the alterations in the osteogenic and apoptotic activity on bone samples from osteoporotic hip fracture patients versus hip osteoarthritis patients. Also, we studied the microstructural and biomechanical characteristics of trabecular bone in these patients.

## Methods

### Patients

The studied population consisted of two groups of patients. The first included 6 patients (1 man and 5 women) with osteoporotic hip fractures (OP), between 62–87 years old and the second one, 6 patients (2 men and 4 women) with hip osteoarthritis (OA), between 55–78 years old. Inclusion criteria in both groups were to be aged 55 or over, not to lead a sedentary life, to walk for 3–4 hours every week, to take more than 700 mg/day of calcium (calculated by dietetic questionnaires) and not receiving treatment with an influence on bone metabolism. The OP group was made up by patients with an osteoporotic hip fracture that required arthroplasty and the OA group by patients with osteoarthritis, admitted for elective hip arthroplasty and not having presented an osteoporotic fracture during their life. Exclusion criteria were malignant diseases, hyperthyroidism, hyperparathyroidism, osteomalacia, previous bisphosphonate or systemic steroid treatment or use of vitamin D supplements or current use of hormone replacement therapy or any other drugs with effects on bone metabolism.

Patients were included in the study in a consecutive manner as they were directed to arthroplasty by the Trauma & Orthopedics Department in the University Hospital “Virgen Macarena” of Seville, Spain.

All patients were included to participle in this study and agreed their bone samples. The study was approved by the Institutional Ethics Committee and written informed consent was obtained from all the participants in the study.

During the surgical procedure, a femoral osteotomy was performed and the femoral head and neck were removed.

### Biochemical parameters

We obtained a fasting blood sample, in the first 48 h after surgery, for analytical determinations of parameters related to bone metabolism: calcium corrected for albumin levels, phosphorus, β- CrossLaps, aminoterminal propeptide of type I procollagen (PINP), parathyroid hormone (PTH), 25-hydroxyvitamin D (25(OH)D) and insulin growth factor-like (IGF-I).

Corrected serum calcium and phosphorus were measured by an autoanalyzer DAX-96.

PTH, β-CrossLaps and PINP were analyzed by immunoassay by an autoanalyzer COBAS 601 (Roche, Spain). Interassay CV <5.8%, <7.6% and <4.2% respectively.

25(OH)D were analyzed by direct competitive immunoassay by an autoanalyzer LIAISON (DiaSorin, Italy). Interassay CV < 5.5%.

Serum IGF-I was measured by immunoassay by IMMULITE (Siemens, Germany). Interassay CV < 3.9%.

### Dual-energy X-ray absorptiometry (DXA)

Bone mineral density (BMD) was measured in the hip and contralateral femoral neck by dual X-ray densitometer with Hologic-DISCOVERY. CV in vivo 2.9% (femoral neck) and 2.5% (total hip).

### Microstructural and biomechanic bone study

The distal femoral region was analyzed without further sample preparation by micro-CT, (SkyScan 1172) at Trabeculae® (San Cibrao das Viñas, Spain). Data sets were rebuilt using a modified Feldkamp algorithm and segmented into binary images (8-bit BMP images) using adaptive local thresholding. Trabecular bone regions were analyzed using the commercial software (Sky-Scan™ CT, 1.6.0) [10].

To evaluate the biomechanical properties we have performed uniaxial compression tests using the IGFA system *(image-guided failure analysis)*. The variables were quantified to describe the mechanical behaviour of bone tissue including the maximum stress (σ), the maximum strain (ε), and the Young’s modulus (E).

### RNA extraction and cDNA synthesis

RNA was isolated from the femurs by using Trizol, following manufacturer’s instructions (Invitrogen, CA, USA).

To carry out the PCR array, total RNA from 6 different patients in each experimental group was pooled (3 μg total) and the pool was reverse-transcribed with RT^2^ First strand kit (SABiosciences, USA) according to manufacturer’s instructions. For the study of OPG and RUNx2 genes, aliquots of RNA (250 ng) were reverse-transcribed with RT^2^ First strand kit (SABiosciences, USA) according to manufacturer’s instructions.

### PCR array and real time PCR

Human osteogenesis and Human apoptosis RT^2^ Profiler^tm^ PCR Arrays (SuperArray, USA) were used to screen the expression of multiple genes in human bone biopsies. They were used according to the manufacturer’s protocol on an ABI Prism 7500 (Applied Biosystems,USA). Results were normalized with the less variable housekeeping gene available in the array, beta-actin, and a significant (p < 0.05). 3–fold change over control was the selected criteria for an experimental gene expression change, similarly to other studies of this kind [11]. Results were analyzed with th RT^2^ Profiler™ PCR Array Data Analysis (http://www.sabiosciences.com/pcrarraydataanalysis.php#Excel).

Duplicate plates were used.

To validate de PCR array, real time PCR was done with cDNA from each patient, and was repeated three times for each gene using SYBRGreen (Roche,) and the primers of Runx2, osteoprotegerin and beta-actin (housekeeping gene) were provided by QuantiTectPrimer Assay (Quiagen,USA). Real-time PCR were performed in the ABI PRISM 7500 system (Applied Biosystems,USA). Results were expressed in mRNA copy numbers, calculated for each sample using the cycle threshold (Ct) value, and normalized against 18S rRNA.

### Statistics

Results were analyzed with th RT^2^ Profiler™ PCR Array Data Analysis (http://www.sabiosciences.com/pcrarraydataanalysis.php#Excel).

Variables were tested for normality of distribution using Kolmogorov-Smirnov test. To statistical significance of the differences between the means of OP and OA gene expression values in the real time PCR analysis were determined by t-Student test. We used the statistical package SPSS 18.0 (Illinois, USA). Statistical differences were set at p < 0.05. Results are presented as mean ± standard error (SEM).

## Results

We analyzed data from 12 patients: 6 with osteoporotic hip fracture (OP) and 6 with hip osteoarthritis (OA), as the control group, who fulfilled the enrolment criteria. Clinical characteristics and laboratory data of the OP and OA groups are summarized in Table 1.

**Table 1:** Anthropometric, DXA and biochemical characteristics of the study population

	**Hip fracture**	**Osteoarthritis**
**N**	**6**	**6**
Age (year)	77.8 ± 4.5	65.4 ± 3.7
Sex (f/m)	5/1	4/2
BMI (body mass index)	29.69 ± 1.5	34.25 ± 2.2
Neck BMD (gHA/cm2)	0.59 ± 0.03	0.74 ± 0.08
Total hip BMD (gHA/cm2)	0.83 ± 0.04	0.88 ± 0.06
25(OH)D (ng/mL)	12.1 ± 2.7	15.4 ± 2.8
P1NP (ng/mL)	41.5 ± 6.04	41.8 ± 11.6
β-CrossLaps (ng/mL)	**0.51** ± **0.1**	0.36± 0.08

f/m . Female/male. BMD, bone mineral density.

Comparasion were assessed by t-Student test. Values are mean ± standard error.

In both groups, the anthropometric and biochemical characteristics were comparable. Although patients with fractures tended to be older than those with OA, age differences did not seem to influence the results because we found no statistical significant differences between groups and also we found no correlation between age and gene expression in either group.

OP patients had lower values of total hip bone mineral density (BMD) (0.83 gHA/cm ^2^ ± 0.04 OP; 0.89 gHA/cm ^2^ ± 0.06 OA) and neck BMD (0.59 gHA/cm ^2^ ± 0.03 OP; 0.74 gHA/OA ± 0.08 cm ^2)^ than the control group, and higher bone remodelling serum marker, β-CrossLaps, 0.51 ng/mL ± 0.1 (OP) vs. 0.36 ng/mL ± 0.08 (OA) without rearching statistical differences. In all patients, the levels of 25 (OH) D were < 20 ng/mL.

### Histomorphometry and biomechanics results

Microstructural indices show a lower bone quality in the group of patients with hip fractures compared to the osteoarthritic group (Table 2).

**Table 2:** Structural data

	**Hip fracture n = 6**	**Osteoarthritis n = 6**	**p**
BV/TV (%)	30.5 ± 2.9	39.4 ± 6.5	**NS**
Tb.Th (mm)	0.2 ± 0.005	0.02 ± 0.02	**NS**
**Tb.Sp (mm)**	0.55 ± 0.02	0.46 ± 0.03	0.04
Tb.N (mm-1)	1.51 ± 0.12	1.90 ± 0.12	0.049
Tb.Pf (mm-1)	**2.25** ± **1.18**	−2.3 ± 2.19	**NS**
SMI	**1.11** ± **0.26**	−0.11 ± 0.74	**NS**
DA	3.38 ± 0.19	2.88 ± 0.34	**NS**
Young’s modulus (Mpa)	406.1 ± 43.1	634.3 ± 161.7	**NS**
σ (Mpa)	8.28 ± 1.21	11.63 ± 2.94	**NS**
ε	0.03 ± 0.005	0.04 ± 0.004	**NS**

Values are mean ± standard error. NS = not statistically significant.

BV/TV: bone volume fraction; Tb.th: trabecular thickness; Tb.Sp: trabecular separation; Tb.N: trabecular number; Tb.Pf: trabecular bone pattern factor; SMI: structure model index; DA: degree of anisotropy; σ: maximum stress; ε: maximum deflection.

We observed significant lower number of trabeculae (Tb.N.) in the bone of hip fracture patients (1.5 mm-1 ± 0.1 vs 1.9 mm-1 ± 0.1; p = 0.049) and furthermore, these patients also have a greater separation between trabeculae (Tb.Sp) (0.55 mm ± 0.46 vs. 0.02 mm ± 0.03, P = 0.04). The thickness of the trabeculae (Tb.Th) was similar in both groups.

The trabecular pattern index (inverse of connectivity index) showed higher values in the fractured patient group. The structure model index (SMI), which implies a relative prevalence of tube-cylinder trabeculae in comparison to plate-form ones, was higher in the group of patients with hip fracture, indicating a greater number of trabeculae in tube-shaped cylinder, less resitant, in these patients.

Biomechanical studies showed that the values of Young’s modulus, maximum stress and maximum deflection, after applying the compression test, were lower in patients with hip fractures compared with osteoarthritic patients, although these differences did not reach significance (Table 2).

### PCR array osteogenesis

We examined whether osteoporotic patients would have any alterations in the osteogenesis in comparison with osteoarthritic patients. If we consider biologically significant variations when the induction or repression of the gene is at least 3 times, we found that 23 genes, from 86 genes involved in osteogenesis, are altered (p <0.05). In most cases, we observed a repression of these genes in osteoporotic compared with the control bone (Table 3). Only FGF1 and VEGFA, genes involved in cell cycle regulation, are overexpressed (3.3 and 3.0-fold, respectively). From the family of metalloproteases, only MMP8 was up-regulated, and it is involved in inflammatory joint processes (5.06-fold).

**Table 3:** Osteogenic genes at least 3-fold upregulated or downregulated in osteoporotic bone tissue

**Gen symbol**	**ID gen(GeneBannk)**	**Function**	**Fold change**
BGLAP	NM_199173	Skeletal Development	- 3.39
BMP5	NM_ 001718	Skeletal Development	- 3.87
BMP6	NM_021073	Skeletal Development	- 3.30
CDH11	NM_001797	Bone Mineral Metabolism	- 3.30
COL14A1	NM_021110	Bone Mineral Metabolism	- 9.54
COMP	**NM_000095**	Bone Mineral Metabolism	- 4.63
EGFR	**NM_005228**	Cell growth and differentiation	−5.28
FGF1	NM_000800	Cell growth and differentiation	3.3
FGFR1	NM_015850	Cell growth and differentiation	- 5.95
FGFR2	**NM_000141**	Cell growth and differentiation	- 5.63
IGF1R	NM_000875	Cell growth and differentiation	- 8.96
VEGFA	NM_003376	Cell growth and differentiation	3.00
TGFBR1	NM_004612	Cell Proliferation	- 6.31
TGFRB2	NM_003242	Cell Proliferation	- 7.04
VCAM1	NM_001078	Cell adhesion Molecules	**- 8.36**
MMP2	NM_004530	Extracellular matrix Proteases	- 4.36
MMP8	NM_002424	Extracellular matrix Proteases	5.06
NFKB1	NM_003998	Transcription Factors and regulators	- 3.06
SMAD2	NM_005901	**Transcription Factors and regulators**	- 3.42
SMAD3	NM_005902	Transcription Factors and regulators	- 5.25
SOX9	NM_000346	Transcription Factors and regulators	- 5.00
RUNX-2	NM_004348	Transcription Factors and regulators	- 2.94
TNF	NM_000594	**Transcription Factors and regulators**	- 9.18

Among the down-regulated genes, it is necessary to emphasize the osteocalcin gene (BGLAP) (− 3.39-fold), a specific gene from osteoblastic cells which function is bone mineralization, CDH11 (−3.3-fold) a typical adhesion molecule of osteoblasts, Runx2 (−2.94-fold) which is a factor of transcription essential for the maturation of osteoblastic cells and BMP6 (−3.3-fold) that encodes a protein of bone formation, also typical from osteoblastic cells. The latter four genes are characteristic of osteoblastic cells and are suppressed on the macerated OP bones.

We also showed down-regulation in several transcription factors such as NFKB1, SMAD2, SMAD3, SOX9 and TNF, which in turn modulate gene expression of several genes.

### PCR array apoptosis

When analyzing array apoptosis, we observed that most of the genes show a decrease in gene expression in the osteoporotic group in comparison with the osteoarthritic group (Table 4).

**Table 4:** Apoptotic genes at least 3-fold upregulated or downregulated in osteoporotic bone tissue

**Gen symbol**	**ID gen(GeneBannk)**	**Function**	**Fold change**
CD27	**NM_001242**	TNF Receptor Family	−6.57
CD40	**NM_001250**	TNF Receptor Family	−7.71
LTBR	NM_002342	TNF Receptor Family	−3.35
TNF	NM_000594	TNF Receptor Family	−16.76
**OPG**	NM_002546	TNF Receptor Family	−3.52
TRAF4	NM_004295	TRAF Family	−3.72
CRADD	NM_003805	CRAD Family	**−4.49**
CASP1	NM_033292	Caspase Family	**−6.31**
CASP4	NM_001225	Caspase Family	−5.34
CASP5	NM_004347	Caspase Family	**−5.93**
**CASP9**	**NM_001229**	Caspase Family	**−6.09**
CASP10	NM_001230	Caspase Family	**−11.93**
APAF1	NM_001160	CARD Family	−2.65
CARD8	NM_0149959	CARD Family	−3.45
NOD1	NM_006092	CARD Family	−3.43
NOL3	NM_ 003946	CARD Family	**−4.89**
RIPK2	NM_003821	CARD Family	−3.96
DAPK1	**NM_004938**	Death Domain Family	−5.92
**BIRC2**	NM_001166	IAP family	−3.22
XIAP	NM_	IAP Family	−4.49
**BAG1**	NM_ 004323	Bcl2-Family	−3.62
BCL2	NM_000633	Bcl2-Family	−6.31
BCL2A1	NM_004049	Bcl2-Family	9.66
BCL2L11	NM_006538	Bcl2-Family	−6.13
BNIP1	NM_001205	Bcl2-Family	2.74
BNIP2	NM_004330	Bcl2-Family	−4.88
ABL1	NM_005157	p53 and DNA damage response	−6.85
BRAF	**NM_004333**	Anti-apoptosis	−6.55

As known, apoptosis can be initiated either by the death receptor-mediated pathway or the mitochondria-mediated pathway. In our study, we found that both pathways are altered on macerated OP bones. Of the 84 genes studied, 26 have a significant lower gene expression in the OP group vs. OA, and only two of them show increased expression (p <0.05). Specifically, we observed a lower expression of TNF receptor family genes as CD40 (−7.7-fold OP vs. OA), LTBR (−3.3-fold), TNFRSF11B (−3.5-fold), CD27 (−6.6-fold) and the TNF ligand Family, TNF (−16.7-fold). At a mitochondrial level, there are repressed genes such as BAD (−5.8-fold) and slightly BAX (−1.2-fold).

Caspases are final effector enzymes of programmed cell death. In the OP group, the caspases studied also have a lower gene expression, observing a significant biologically variation for caspase 9 (−6.1-fold OP vs. OA) and caspases 4 and 5 (−5.3- fold and −5.9-fold-respectively).

We have also observed the repression of genes such as Bcl2 (−6.3-fold) and BAG1 (−3.62-fold), both of the bcl-2 family and ABL1 (−6.8-fold) involved in the damage response DNA.

In OP patients, we only observed an increased in the gene expressions of BCL2A1 (9.66-fold) and BNIP1 (2.7-fold), both from the BCL2 family of anti-apoptotic features.

To verify the results obtained in the array plates, we chose two genes, with particular relevance to the activity of bone remodelling, such as Runx2 and OPG. Runx2 is the major transcription factor involved in osteoblast differentiation, and the results of the osteogenesis array plate indicate a down-regulation in OP vs. OA bone of this gene (−2.94-fold, p <0.05) (Table 3). OPG is part of one of the main systems of regulation of bone remodelling (OPG/RANKL/RANK) and the array plate are decreased apoptosis expression in the OP group compared with the OA group (−3.52-fold, p < 0.05) (Table 4).

**Figure 1:**
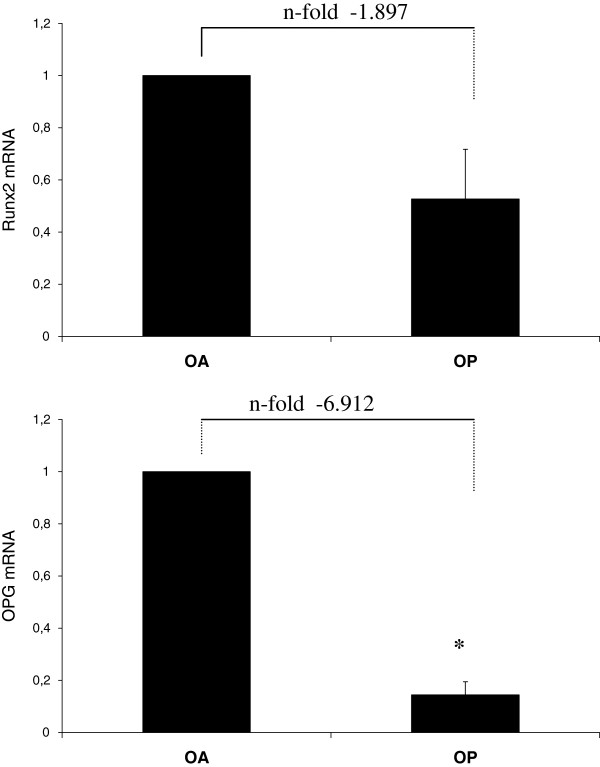
**Normalized mRNA levels for Runx2 and OPG in human femur of patients with OP and OA**. Results are mean ± S.E.M: (n = 3). The results reported in percentages of the variation referred to OA group. *p < 0.05 OP vs. OA.

When analyzed individually OPG and Runx2 gene, we observed that the OP group has almost 2 times less expression of Runx2 and 6.9 times less OPG than the control group (p = 0.004), confirming the results of osteogenesis and apoptosis arrays (Figure 1).

## Discussion

The osteogenic activity is mediated by multiple proteins and may be altered by different circumstances. We investigated whether genes associated with this activity may have a different expression in bone tissue of osteoporotic fracture patients versus osteoarthritis patients. We have studied 86 genes related to osteogenic activity and 84 to apoptosis. Most of them have a lower expression in osteoporotic bone than in osteoarthritic bone, indicating that the bone tissue in osteoporosis is more inert, having less transcriptional activity.

Regarding osteogenic activity, the macerated OP hip bone has repressed typical genes of osteoblast cell. So BGLAP (essential for bone mineralization), CDH11 (osteoblast adhesion molecule) and Runx2 (transcription factor required for osteoblastogenesis), have a lower expression in osteoporotic than arthritic bone, which is consistent with decreased osteoblast differentiation and activity in osteoporosis bone. This finding agrees with previous results [12,13], and low levels of osteocalcin published by Napal et al. in stressful situation [14]. These results were confirmed by the analysis of the expression of Runx-2 by real-time PCR, significantly lower in samples from OP patients, in comparison with samples from OA patients.

Several TGF-β/BMP signalling pathway components and modulator genes that influence osteoblast function, bone remodelling and bone mineralization, were down-regulated in OP bone, such as TGFBR1, TGFBR2, BMP5, BMP6, SMAD 2 and SMAD3. Several authors agree with these results, and it is consistent with decreased in the bone turnover in OP bone [15-18].

Previous studies demonstrated that BMP6 was more potent and consistent than BMP2 and BMP7 in inducing osteoblast differentiation in primary hMSC [19], so that a decrease in the expression of BMP6 in OP bone indicates a minor osteoblast differentiation. In the other hand, BMP5 accelerates expression of hypertrophy markers which is of relevance in both development and diseases such as osteoarthritis [20], this is consistent with the presence of higher values in OA patients macerates against OP ones.

With regard to genes encoding the synthesis of metalloproteinases, we have also found differences when comparing the osteoporotic and arthrosic bone. The lack of MMP8 is accompanied by synovial inflammation and bone erosion in mice arthritis model, indicating that MMP8 has a protective role in this disease. The fact of finding higher expression levels MMP8 in OP patients than OA may suggest an increased activity in inflammatory joint in OA patients [21,22].

In cell cycle regulation, multiple receptors and modulators of different activation pathways are involved. Among the genes studied in this group, we obtained a decreased gene expression of EGFR, FGFR1, FGFR2 and IGF1R in the OP group macerates. On the contrary, FGF1 and VEGFA are two of the three genes with an increased expression in the OP group.

Epidermal growth factor receptor (EGFR) signalling pathway is an important bone regulator and it primarily plays an anabolic role in bone metabolism. A physiological function of osteoblastic EGFR signalling is maintaining the pool of osteoprogenitors at an undifferentiated stage in bone. It is reported that blocking EGFR activity in osteoblast lineage cells results in fewer osteoprogenitors and leads to defective bone formation [23]. Fibroblast growth factors (FGFs) are polypeptides that control the proliferation and differentiation of various cell types including osteoblasts. FGFs are also strong inducers of angiogenesis, necessary to obtain oxygen and nutrients during tissue repair [24]. Some authors have described that the administration of FGF1 increases new bone formation and bone density [25]. Our findings about a minor expression of receptors in OP bone indicate a reduced capacity for bone regeneration in these patients, which tries to compensate with increased expression of the ligand FGF1. We also found high levels of VEGF in OP bone as other authors who described an increase of mRNA expression of VEGF in femoral neck fracture versus nontraumatic femoral head in humans [26].

All these results taken together suggest that osteoblastic activity is decreased in osteoporotic bone and bone formation rate is lower in OP than in OA bone.

The human apoptosis PCR array profiles the expression of 84 key genes involved in apoptosis or programmed cell death. We have observed a down-regulation in 26 genes and upregulation in only two of them.

The two major pathways of apoptosis are the extrinsic (Fas and other TNFR superfamily members and ligands) and the intrinsic (mitochondria-associated) pathways [27]. We found no differences in the expression of genes related to the FasL family but we showed a lower expression of genes of the TNF family in OP bone, such as CD27, CD40, LTBR, TNF and OPG. CD27 and CD 40 are essential in mediating a broad variety of immune and inflammatory responses [28], consistent with their increased gene expression in OA patients rather than in OP patients.

Caspases are proteases that cleave intracellular proteins and are required to carry out apoptosis [29]. In OP group, we found a down-regulation in all caspases studied, being biologically significant decreases in the expression of caspases −1, -4, -5, -9 and −10, indicating that in the OP group there is a clear decrease in apoptotic activity. The activation of these caspases requires binding to specific cofactors, caspases possess different domains, containing death-effector domains (DED) or caspase recruitment domains (CARD) [30] and we also observed a down-regulation in some of these cofactors, specifically with Apaf-1, CARD8, NOD1, NOL3, RIPK2 and DAPK1, in OP bone.

Bcl-2 proteins are a family of cytosolic proteins involved in the mitochondrial pathway of apoptotic and these can be either anti or pro-apoptotic. They act as a sensor of cellular damage. At present, regulation of apoptosis by Bcl-2 family members is complex and not fully understood because most cells express a variety of antiapoptotic and proapoptotic Bcl-2 proteins, and the regulation of their interactions dictates survival or commitment to apoptosis [31]. Our studies show a decrease in the expression of members of the Bcl-2 anti-apoptotic family such as BAG1, BCL2 (one of the most important and well known), BCL2L11 and OP BNIP2 in bone and increased the expression of BCL2L11 and BNIP1. We suggest that the balance of the expression of these genes can lead different cells in the osteoporotic bone towards apoptosis inhibition. Although, we were waiting high levels expression of caspases, they found low, this might be due to the differences type of cells in the bone samples (osteoblasts, osteocytes, mesenchymal stem cells …). Some authors described increased apoptosis of osteocytes after an osteoporotic fracture [32], but the apoptosis of osteoblasts was not altered. Furthermore, it seems that an increase in apoptosis indicates an increase of bone remodeling, and we observed an osteoporotic bone with low transcription level and consequently with low bone remodelling [8].

OPG plays essential roles in the osteoblast cell, on the one hand it inhibits bone resorption, joining the receptor activator for nuclear factor _B ligand (RANKL) [33] and on the other hand, it can also bind and antagonize the activity of the TNF-related apoptosis-inducing ligand (TRAIL) [34]. We found decreased levels of OPG expression in OP bone, in PCR array and also to check real-time PCR.

The histomorphometry data obtained indicate that OP bone is of lower quality than OA, observing a smaller number of trabeculae, greater separation between them and poorer connectivity [35]. These characteristics indicate that OP bone is more fragile and such fragility is determined by a worse bone structure. We could not correlate these values with any of the genes studied, although some authors suggest a relationship between low expression of Runx2, in biopsies from the iliac crest from patients with male idiophatic osteoporosis, and a significant decrease in the number of trabeculae and conectivity density [36], data that support our findings.

Our study has some limitations. Firstly, we studied trabecular bone, which may behave differently of cortical bone and secondly we present a relatively low number of patients.

These results open a wide field of research in the treatment of osteoporotic and arthritic disease. Noting the different gene activities that are in both conditions, we can deepen the study of each of these genes looking for the best candidate as a therapeutic target. In our work, we point out the genes with differential expression and also we relate gene activity to structural and biochemical differences in bone.

## Conclusions

In summary, our findings lead us to conclude that bone tissue of osteoporotic fracture patients presented worse microstructural characteristics which make them more fragile than the bone of OA patients, in line with other authors [35]. This may be related to an imbalance in bone remodelling that is conditioned by a lower expression of genes encoding proteins that promote osteogenic activity. The result is a bone with a lower capacity for differentiation and osteoblastic activity as well as decreased activity of bone remodelling and mineralization capacity. This bone also shows data suggesting a lower rate of apoptosis.

## Abbreviations

OP: Osteoporosis;OA: Osteoarthritis;OPG: Osteoprotegerin;RANKL: Receptor activator for nuclear factor _B ligand;BMD: Bone mineral density;Tb.N: Number of trabeculae;Tb.Sp: Separation between trabeculae;Tb.Th: Thickness of the trabeculae;SMI: Structure model index;BGLAP: Osteocalcin gene;EGFR: Epidermal growth factor receptor;FGF: Fibroblast growth factor

## Competing interests

The authors declare that they have no competing interests.

## Authors’ contributions

MG carried out the molecular genetic studies, participated in the bioinformatics analysis and drafted the manuscript. MJM and MAV participated in the clinical and biochemical analysis. CM carried out the bioinformatics analysis. RPC conceived of the study, and participated in its design and coordination and helped to draft the manuscript. All authors read and approved the final manuscript.

## Pre-publication history

The pre-publication history for this paper can be accessed here:

http://www.biomedcentral.com/1471-2474/14/41/prepub
